# A Personalized User Authentication System Based on EEG Signals

**DOI:** 10.3390/s22186929

**Published:** 2022-09-13

**Authors:** Christos Stergiadis, Vasiliki-Despoina Kostaridou, Simos Veloudis, Dimitrios Kazis, Manousos A. Klados

**Affiliations:** 1 Department of Psychology, City College, University of York Europe Campus, 54622 Thessaloniki, Greece; 2 Neuroscience Research Center (NEUREC), City College, University of York Europe Campus, 54622 Thessaloniki, Greece; 3 Department of Computer Science, City College, University of York Europe Campus, 54622 Thessaloniki, Greece; 4 3rd Department of Neurology, Aristotle University of Thessaloniki, Exochi, 57010 Thessaloniki, Greece

**Keywords:** biometrics, EEG, security, user authentication, machine learning, applied neuroscience

## Abstract

Conventional biometrics have been employed in high-security user-authentication systems for over 20 years now. However, some of these modalities face low-security issues in common practice. Brainwave-based user authentication has emerged as a promising alternative method, as it overcomes some of these drawbacks and allows for continuous user authentication. In the present study, we address the problem of individual user variability, by proposing a data-driven Electroencephalography (EEG)-based authentication method. We introduce machine learning techniques, in order to reveal the optimal classification algorithm that best fits the data of each individual user, in a fast and efficient manner. A set of 15 power spectral features (delta, theta, lower alpha, higher alpha, and alpha) is extracted from three EEG channels. The results show that our approach can reliably grant or deny access to the user (mean accuracy of 95.6%), while at the same time poses a viable option for real-time applications, as the total time of the training procedure was kept under one minute.

## 1. Introduction

As modern society has already transitioned into the era of information and technology, security and privacy are becoming increasingly important. The need for effective user authentication systems in a trusted and autonomous manner is more evident than ever, in order to prevent intruder attacks or information leaks. The potential of using Electroencephalography (EEG) signals as a biometric tool for person authentication is an area that has attracted increased attention over the last decade [1,2,3,4,5,6,7,8,9,10,11,12], as it overcomes some of the deficiencies that the already-established methods do present, such as ensuring the liveliness of the user and allowing for continuous user authentication [13].

Biometrics, in general, are defined as the unique behavioral or physiological characteristics that can be used for the identification of a person [14]. Authentication systems using well-established biometric signals such as fingerprint, iris, voice, and gait recognition regularly face low-security issues, as they are vulnerable to spoofing tools [5]. Such tools can be artificially generated “gummy” fingers [15] for fooling fingerprint recognition, voice coders for voice recognition, contact lenses for iris recognition, and adversarial attacks for gait recognition [16]. In addition to these issues, conventional biometric-based authentication systems may give rise to violent attacks in which the attacker forces the victim to provide his/her biometric traits to the system (e.g., at a gunpoint threat, using a dismembered finger, etc.). Other means of authentication, such as password-based techniques (something that the person knows), may be easy to use but are also threatened by malicious attacks, such as the popular dictionary or brute force attack, and the exploitation of user mistakes [13], while also being vulnerable to social-engineering related attacks. Additionally, token-based authentication, which is connected to something that the user possesses, for example, a key, a card, or a USB Dongle [17], can be proven inconvenient as the user needs to carry the token every time that he/she requires access. Moreover, there is also the danger of the object being stolen or mimicked by reverse-engineering techniques [17].

A user authentication method based on brainwave activity can address the aforementioned drawbacks, or complement them, and also provide solutions to high-level security systems and systems with continuous authentication requirements [18]. Electroencephalography (EEG) is a highly individualistic biometric that has high inter-subject variability and low intra-subject variability [12]. Therefore, it can be used for the efficient identification and authentication of a user and ensure shielding against intruders. Moreover, brainwaves are an intrinsic characteristic that makes EEG strong against mimicking and identity theft, in contrast with conventional biometrics stemming from the human body. In fact, an attacker cannot force the user to authenticate himself, as stress and pressure seriously affect the EEG signals [5], while the liveliness of the user is also ensured. Another important key factor of EEG is that it features cognitive processes that can be detected unconsciously, providing the opportunity for continuous user authentication.

During the last decade, a large number of publications have emerged, dealing with EEG-based authentication techniques [1,2,3,4,5,6,7,8,9,10,11,12]. These works naturally strive to optimize the accuracy and ease of use of the proposed approaches by typically relying on an efficient combination of the EEG features that are chosen to represent the individual’s brain activity on one hand, and the classifier that is used for the classification and the final decision of the system to grant or deny access on the other. Pham et al. [13] used Autoregressive (AR) linear parameters and Power Spectral Density (PSD) components (3–80 Hz), which were fed into a Support Vector Machine (SVM) classifier, achieving an Equal Error Rate (EER) as low as 0.002. The same authors later investigated the same combination in a different frequency band (1–30 Hz) achieving an authentication system with 97% accuracy [19].

Both AR and PSD are widely used in EEG authentication studies. Poulos et al. [20] combined AR features with Kohonen’s Vector Quantizer (VQ) as the classifier, while Paranjape et al. [21] classified the same type of features with discriminant analysis algorithms. In more recent studies, Thomas et al. [22] experimented with resting-state Eyes Open (EO) and Eyes Closed (EC) EEG, extracting PSD features in the gamma band (30–50 Hz) and used specific thresholds in order to grant (or deny) access to the user, finally achieving EER = 0.019. Additionally, in many studies, external stimuli or specific imaginary tasks were presented to the user, and the utility of specific features of the Event-Related Potentials (ERPs) was assessed. For example, Valsaraj et al. [14] analyzed EEG data, looking for characteristic features in the ERPs that were elicited from Motor Imagery (MI) and real movements. By combining different MI actions and using AR and PSD, their proposed authentication method reached an accuracy of 98.28%.

Another direction of research in this field uses the Neural Network (NN) classifier for human identification and classification. From very early-stage studies, Poulos et al. [23] employed the learning vector quantizer (LVQ) and spectral features reaching accuracies ranging from 80% to 100%. Subsequently, the same researchers experimented on the same classifier but with AR and bilinear model features [24], resulting in accuracies from 56% to 80%.

Later on, the back-propagation and the feed-forward Neural Networks gained prominence, with Hema and Osman [25] achieving average accuracies between 80% and 90% by using PSD features and feed-forward NN for their classification. Mu and Hu [26] reached 80% accuracy in their authentication system by choosing AR and Fischer distance as the features and a back-propagation (BP) NN classifier. The Fischer distance was also drafted in later studies, along with fuzzy entropy as the representative features when visual stimuli (self-photos vs. non-self-photos) were introduced to the subject [5], and with the use of BP Neural Network as the classification theme, accuracies of 87.30% were achieved, with a False Acceptance Rate (FAR) of 5.50% and a False Rejection Rate (FRR) of 5.60%.

The most recent studies in the area of EEG biometrics started utilizing novel methods for optimizing the efficacy and reliability of the authentication system. Damaševicius [27] proposed an EEG-based cryptographic authentication system, based on the fuzzy commitment scheme and error-correcting Bose–Chaudhuri–Hocquenghem (BCH) codes. By following a three-step cryptographic procedure (Encoding–Enrollment–Authentication), and by using a cryptographic key and a number of codewords, their biometric cryptosystem achieved an EER of 0.024, with TAR being 0.99. Breakthroughs in the area of deep learning EEG authentication have also been proposed, as such techniques are often susceptible to capturing recording-specific features rather than individual neural biomarkers [28], despite their overall great usability. An answer to this problem was provided by Ozdenizci [28], who introduced an adversarial inference approach for extracting session-invariant person-discriminative features from the data, achieving up to 72% accuracy in across-session person identification. Finally, the issue of EEG channel selection was targeted, as the more electrodes used, the more complex the system becomes and the higher the discomfort for the person to be authenticated. In this line, Alyasseri [29] approached the EEG channel selection as an optimization problem, employing a binary version of the Grey Wolf Optimizer (BGWO), which is a powerful metaheuristic swarm-based algorithm, together with an SVM classifier. Their method managed to reduce the total number of channels from 64 to 23, and with this setup, the classifier achieved 94.13% accuracy (however, this method was used for identification rather than authentication of a user).

Most of the current works have chosen a specific combination of targeted features and a classifier in order to complete their analysis and achieve optimal accuracy. Nevertheless, none of the proposed methods are user-specific, nor do they take into consideration the specific characteristics, each time, of the given dataset. This may reduce the performance of the implemented analysis and affect the classification accuracy. Hence, a practical method that addresses this problem needs to be devised if we want to consider EEG signals as a viable option for real-time human authentication, with advanced accuracy and reliability. The present study proposes an EEG-based authentication system built around the spectral features that characterize brain activity, and the implementation of a Machine Learning (ML) algorithm for the classification of these features. Auto-WEKA software [30] is used in order to ensure that the used algorithms are the most suitable for our data. More specifically, 15 power spectral density features are extracted from three central electrodes (Fz, Cz, Pz) in five different frequency bands, for 15 subjects. The choice for the best descriptive features, as well as the optimal choice of the ML algorithm, is then automatically appointed by running Auto-WEKA.

The rest of the paper is organized as follows. Section 2 presents the dataset, equipment, features, and classification methods used in the study. In Section 3, we present the results for the 15 subjects tested herein, while in Section 4, we provide a discussion of our findings in light of the current literature and a conclusion of our work.

## 2. Materials and Methods

### 2.1. Participants

The sample of participants consisted of 15 individuals, and more specifically, 8 males and 7 females with a mean age of 23.2 ± 5.5 for the males and 21.2 ± 3.4 for the females. The criteria that were set for exclusion from participation concerned the history of neurological and psychiatric illness, substance abuse history, medication, and any other coexisting factors that could affect the brain’s neurophysiology. All participants had normal or corrected to normal vision and were asked to not consume alcohol or caffeine the day prior to their participation. All the experiments were performed at the same time, to the best possible extent. This research was approved by the Ethical Committee of Aston University. Participation was anonymous and confidential.

### 2.2. EEG Data Acquisition

EEG measurements were recorded from 128 electrodes distributed across the scalp according to the EGI Geodesic EEG System (GES) and with reference electrodes positioned at the mastoids. The correct EEG Geodesic net size was soaked in a saline solution for 5 min and applied to their head. The recordings were performed with an EGI GES 300 system. All electrode impedances were maintained at less than 5 kΩ. The sampling rate for all measurements was 250 Hz. After each experiment, the nets were disinfected in order to maintain high hygienic standards. The data used for the purposes of this work were recorded during a resting state with their eyes opened (30 s where participants were asked to keep their eyes open and fixate on a cross appearing at the center of the screen) in order to simulate a real scenario of user identification, and only Fz, Cz, and Pz were further used.

### 2.3. Preprocessing and Feature Extraction

All EEG signals were referenced according to the linked earlobe montage [31], filtered at the frequency range of 0.5–40 Hz, and submitted to the Adaptive Mixture ICA (AMICA) algorithm [32], and the REG ICA [33,34] methodology was used to clean the independent components from artifacts. Specifically ocular artifacts, which are the most troublesome in eyes-open conditions, were cross-validated using the EEGLAB’s [35] toolbox called ICLABEL [36].

The cleaned EEG signals were divided into 500 random segments of 4 s each, in order to increase the external validity of the current study. For each segment, the power of five different brain waves was computed (delta [0–4 Hz], theta [4–8 Hz], lower alpha [8–10 Hz], higher alpha [10–12 Hz], and alpha [8–12 Hz]). This resulted in a feature-set of 15 (3 channels × 5 bands) features per subject with 500 instances.

### 2.4. Classification Procedure

**Figure 1:**
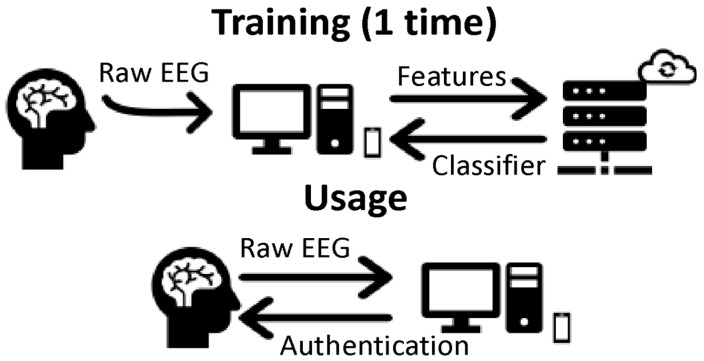
System illustration of the two different phases of the classifier. First, there is a training phase where the server computes the optimal classification algorithm, and then there is a usage phase (test phase) where the system grants or denies access to the user based on this classifier.

In order to replicate a realistic scenario, classification was performed on an individual basis. Thus, for each participant (user), a separate dataset of 1000 instances was formed, where half of the instances were derived from the 500 random segments from the user and the other 500 were randomly chosen from the remaining 14 participants. In an ideal scenario, a robust user authentication algorithm should grant access to the 500 instances that come from the user and deny access to the remaining 500 instances coming from the other 14 users. This procedure resulted in 15 different user-specific datasets that were further imported into WEKA [37], where Auto-WEKA [30] was used, with 1 min as the time limit for training. The default time limit was reduced from 15 min to only 1 min keeping in mind the usability of our approach (Figure 1). The accuracy was computed using the 10-fold cross-validation scheme, which means that the data were divided into 10 random subsets of 100 instances. Nine of these were used as the training set and one as the testing set. After the development of the classifier, its accuracy was defined by the correct classification of the test set’s instances. This procedure was repeated 10 times in order to account for the sampling bias. Each time, a new test set was used, and the overall accuracy was determined by averaging the accuracies found. 

### 2.5. Evaluation

The evaluation of the proposed methodology was based on three classical measurements in the area of EEG biometrics, namely the False Acceptance Rate (FAR), the False Rejection Rate (FRR), and the Equal Error Rate (EER). The classification accuracy will also be reported and compared to previous works. The EER is defined as the point where the FAR and the FRR become equal. The lower the value of the EER, the more accurate the classification. So, mathematically:

EER = FAR(T*) = FRR(T*)

where T* = arg min((|FAR(T)-FRR(T)|).

In the proposed system, the user wears a wireless and wearable EEG headset that sends the raw EEG signals (~30 s) to the terminal authentication app. The app computes the 15 features with 500 instances mentioned above, which are uploaded to the server. The server forms a dataset consisting of 500 instances of the user and a random selection of another 500 instances from other users that are stored in the database. Then, the server computes the optimal, user-specific, classification algorithm and returns it to the terminal app. The aforementioned procedure lasts approximately 1 min and runs only once (upper schema). Since the classifier is saved in the terminal app, the app can grant or deny access to the user using the provided EEG data.

## 3. Results

Table 1 presents the authentication results after the proposed method was implemented on the 15 subjects. Mean accuracy of 95.6% was obtained for all subjects. In 13 out of the 15 subjects, the accuracy was above 94%, promoting the high level of performance of the system. The highest accuracy was obtained for the first subject (100%), while the lowest levels of accuracy were observed for the fourth subject (87%), which seems to be an outlier and indicates that much better overall results could be obtained if we excluded this subject from our analysis. 

**Figure 2:**
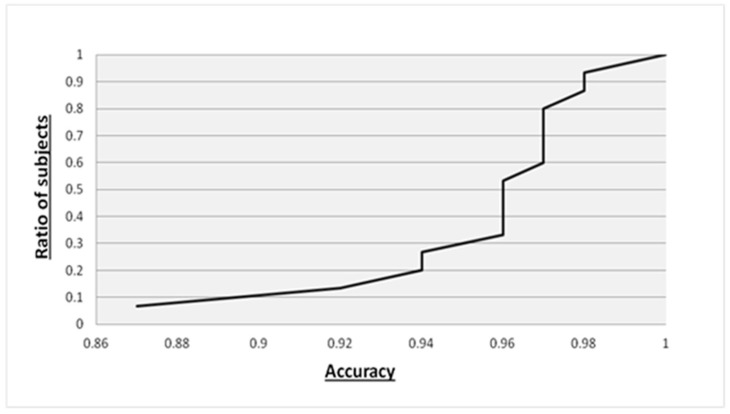
Cumulative distribution plot of accuracy distribution in subject classification.

This observation was statistically tested with the Shapiro–Wilk normality test, as the data went from non-normally distributed (W = 0.84, *p* = 0.01) to normally distributed (W = 0.93, *p* = 0.41) after excluding the results for subject 14. Nevertheless, by inspecting the cumulative distribution plot of the accuracy distribution of all 15 patients (Figure 2), we can observe that in 50% of the subjects, the accuracy is above 96%, while only 10% of the subjects have accuracies lower than 90%.

**Figure 3:**
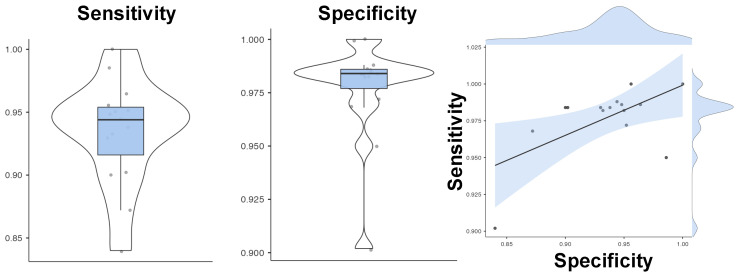
Classification performance. The violin and the box plots describe the distribution of sensitivity and specificity across the 15 subjects, while from the scatterplot, it is obvious that one subject was an outlier indicating that our results would be much better by excluding this subject.

The mean sensitivity of the system, which is expressed by the means of the True Positive Rate (TAR), was 0.93 (±0.04). For TAR, we define the ratio of the user’s instances that were correctly given access to the system against the total number of instances (500). In addition, the specificity of our proposed system, which is assessed through the True Negative Rate (TRR), was 0.98 (±0.02). The TRR is the number of intruder instances that are correctly denied by the system against the total 500 instances, forbidding access to the individual. In Figure 3 you can see the distributions of both sensitivity and specificity, as well as their relationship. 

Furthermore, we employed statistical analysis, despite the very obvious results, in order to prove the efficacy of our approach statistically. The accuracy, FAR, and FRR were tested using a one-sample t-test or a one-sample Wilcoxon W test when the variables were not normally distributed. According to the Shapiro–Wilk test of normality, only FAR is normally distributed (W = 0.944, *p* = 0.437), while accuracy (W = 0.835, *p* = 0.011) and FRR (W = 0.705, *p* = 0.001) are not normally distributed. So, for FAR, the one-sample t-test revealed that our approach has significantly lower FAR (M = 0.06, SD = 0.04) than random guessing, t(14) = −40.4, *p* = 0.001. On the other hand, the W test for accuracy revealed that our approach has significantly higher accuracy (M = 95.6%, SD = 2.9%) than random guessing W(14) = 120, *p* = 0.001, while FRR was significantly lower (M = 0.02, SD = 0.01) than random guessing W(14) = 0, *p* = 0.001. The mean Equal Error Rate (EER) of the system, across the 15 subjects, was found to be equal to 0.064.

## 4. Discussion and Conclusions

The usage of EEG signals in human authentication systems has proven to be a very effective technique as it overcomes most of the drawbacks that conventional biometric tools, such as iris, fingerprint, or voice-based applications, present in everyday practice. Some of the advantages of using brainwaves include their suitability for continuous user authentication systems, as they pose nonconscious biometrics, and the fact that they can be utilized in high-level security facilities, ensuring the liveliness of the person requiring access and averting intruder attacks.

Despite the increasing number of studies in this field and the numerous different approaches (Table 2), from trying to find the optimal combination of features (AR models, PSD components, etc.) and classification algorithms (LDA, SVM, CNN, etc.), to the use of innovative measures such as fuzzy entropy and exploiting eye blinking signals, no studies, to the best of our knowledge, have taken into account the individual characteristics of the dataset in use. In this paper, we computed the PSD of the EEG signal in five different bands (delta [0–4 Hz], theta [4–8 Hz], lower alpha [8–10 Hz], higher alpha [10–12 Hz], and alpha [8–12 Hz]) from three central electrodes (Fz, Pz, and Cz) resulting in 15 different features for each subject.

The novelty of our method is that we introduced the use of the machine learning algorithm “WEKA” as an extra step after the feature extraction stage, in order to appoint the optimal feature set and classification algorithm for each individual. As a result, we achieved an overall mean accuracy of 95.6%, with a mean FAR of 0.023, a mean FRR of 0.065, and an EER of 0.064. Another important feature of the proposed methodology is that the EEG signal was recorded from only three central electrodes and for just 30 s, making the system very efficient. In addition, the total time for the EEG signal recording, feature extraction, and auto-WEKA algorithm selection was kept under one minute. This study reflects the limitations and implications of real-life practices and aims to provide solutions for the practical use of such EEG-based authentication systems. 

In order for our report to be comprehensive, a comparison with the rapidly growing deep learning authentication techniques would be fruitful. Deep learning models have the advantage of reliably capturing the high-dimensional feature representation of the signals and the possible relationship between internal features through the non-linear deep structure [40]. Another great advantage of deep learning techniques is that they can be performed based only on the original data, bypassing the complex pre-processing stage and feature extraction processes. Considering accuracy, deep learning methods can reach some outstanding performances. For example, Mao [41] achieved 97% accuracy by feeding the raw EEG data of 100 subjects into a CNN. However, deep learning models tend to have much higher computational costs than simpler machine learning ones. For example, in his model, although achieving an accuracy of 91.44%, a previous author [42] had an average time of 28.5 minutes for implementing their deep-learning-based authentication technique. In real-life applications, such time slots are prohibitive. In comparison, our proposed method poses a very reliable alternative, as it combines a mean accuracy of 95.6% with an overall running time of 60 s.

Finally, regarding future directions, EEG-based user authentication has some general issues that need to be addressed. For example, if the user is not interested in being authenticated, then his/her brainwaves can be altered, leading to failures of the authentication system. Furthermore, regarding our approach and proposed methodology, further datasets need to be examined, and more specifically, a larger number of participants need to be tested on the system, as the rather limited number of subjects (15) in the present study poses aa problem for safely generalizing our results, threatening the external validity of the study. At last, the fact that we only assessed spectral features in our study may not fully exploit the capabilities of the personalized user authentication system, the implementation of which may be further refined by the introduction of hybrid spectral-time domain features. The possibility of using some of the recently emerged and increasingly used deep learning algorithms, together with the advancements that have been introduced for their refinement such as the adversarial inference approach [28], can also provide higher accuracies despite the likely increased computational cost.

## Figures and Tables

**Table 1:** Authentication results. The classification accuracy, sensitivity, and specificity across the 15 subjects for the 500 instances coming from the EEG of each user and the 500 randomly chosen instances coming from the remaining 14 subjects, which should be treated as the impostor signals.

	GRANT ACCESS		DENY ACCESS		Sensitivity		Specificity		
SUBJECT	GRANT	DENY	GRANT	DENY	TAR	FAR	TRR	FRR	Accuracy
SS01	500	0	0	500	1	0	1	0	100%
SS02	436	64	16	484	0.872	0.032	0.968	0.128	92%
SS03	493	7	25	475	0.986	0.05	0.95	0.014	97%
SS04	420	80	49	451	0.84	0.098	0.902	0.16	87%
SS05	474	26	7	493	0.948	0.014	0.986	0.052	97%
SS06	450	50	8	492	0.9	0.016	0.984	0.1	94%
SS07	475	25	9	491	0.95	0.018	0.982	0.05	97%
SS08	476	24	14	486	0.952	0.028	0.972	0.048	96%
SS09	469	31	8	492	0.938	0.016	0.984	0.062	96%
SS10	482	18	7	493	0.964	0.014	0.986	0.036	98%
SS11	466	34	9	491	0.932	0.018	0.982	0.068	96%
SS12	472	28	6	494	0.944	0.012	0.988	0.056	97%
SS13	465	35	8	492	0.93	0.016	0.984	0.07	96%
SS14	478	22	0	500	0.956	0	1	0.044	98%
SS15	451	49	8	492	0.902	0.016	0.984	0.098	94%

**Table 2:** Previous EEG-based user authentication studies.

Paper	No. of Subjects	No. of EEG Channels	Features	Accuracy
[19]	40	8	AR linear parameters and PSD components (1–30 Hz)	97.10%
[38]	10	2 (Fp1 & Fp2)	Fuzzy entropy and Fisher distance	87.30%
[12]	8	9	Low-frequency SSVEP components	96.78%
[39]	10	10	Wavelet Packet Decomposition and Correlation-based features	95%
[3]	32	1	Wavelet based (time-frequency) features: (1) mean (2) standard deviation (3) entropy for the wavelets of the five frequency bands	94.04%
[14]	25	4	AR linear parameters, PSD components	98.28%
[1]	5	14	(1) AR coefficients (2) PSD components (3) total power (4) interhemispheric power differences (5) interhemispheric linear complexity	97.69%
[11]	10	18 + 5 subject-specific channels	The difference between the averaged signals in response to self-face	86.10%

## Data Availability

The data presented in this study and the implementation source code are available on request from the corresponding authors.

## References

[1] Ashby C., Bhatia A., Tenore F., Vogelstein J. Low-Cost Electroencephalogram (EEG) Based Authentication. Proceedings of the 2011 5th International IEEE/EMBS Conference on Neural Engineering.

[2] Chuang J., Nguyen H., Wang C., Johnson B., Adams A.A., Brenner M., Smith M. (2013). I Think, Therefore I Am: Usability and Security of Authentication Using Brainwaves. Proceedings of the Financial Cryptography and Data Security.

[3] Gui Q., Jin Z., Xu W. Exploring EEG-Based Biometrics for User Identification and Authentication. Proceedings of the 2014 IEEE Signal Processing in Medicine and Biology Symposium (SPMB).

[4] Jayarathne I., Cohen M., Amarakeerthi S. BrainID: Development of an EEG-Based Biometric Authentication System. Proceedings of the 2016 IEEE 7th Annual Information Technology, Electronics and Mobile Communication Conference (IEMCON).

[5] Abo-Zahhad M., Ahmed S.M., Abbas S.N. (2016). A New Multi-Level Approach to EEG Based Human Authentication Using Eye Blinking. Pattern Recognit. Lett..

[6] Nguyen B., Nguyen D., Ma W., Tran D. Investigating the Possibility of Applying EEG Lossy Compression to EEG-Based User Authentication. Proceedings of the 2017 International Joint Conference on Neural Networks (IJCNN).

[7] Pham T., Ma W., Tran D., Nguyen P., Phung D., de la Puerta J.G., Ferreira I.G., Bringas P.G., Klett F., Abraham A., de Carvalho A.C.P.L.F., Herrero Á., Baruque B., Quintián H., Corchado E. (2014). EEG-Based User Authentication Using Artifacts. Proceedings of the International Joint Conference SOCO’14-CISIS’14-ICEUTE’14.

[8] Phothisonothai M. An Investigation of Using SSVEP for EEG-Based User Authentication System. Proceedings of the 2015 Asia-Pacific Signal and Information Processing Association Annual Summit and Conference (APSIPA).

[9] Wong R.-Z., Choo Y.-H., Muda A.K. (2020). Task Sensitivity in Continuous Electroencephalogram Person Authentication. Int. J. Adv. Comput. Sci. Appl. IJACSA.

[10] Wu Q., Zeng Y., Zhang C., Tong L., Yan B. (2018). An EEG-Based Person Authentication System with Open-Set Capability Combining Eye Blinking Signals. Sensors.

[11] Yeom S.-K., Suk H.-I., Lee S.-W. (2013). Person Authentication from Neural Activity of Face-Specific Visual Self-Representation. Pattern Recognit..

[12] Yu T., Wei C.-S., Chiang K.-J., Nakanishi M., Jung T.-P. EEG-Based User Authentication Using a Convolutional Neural Network. Proceedings of the 2019 9th International IEEE/EMBS Conference on Neural Engineering (NER).

[13] Pham T., Ma W., Tran D., Nguyen P., Phung D., Motoda H., Wu Z., Cao L., Zaiane O., Yao M., Wang W. (2013). EEG-Based User Authentication in Multilevel Security Systems. Proceedings of the Advanced Data Mining and Applications.

[14] Valsaraj A., Madala I., Garg N., Patil M., Baths V. Motor Imagery Based Multimodal Biometric User Authentication System Using EEG. Proceedings of the 2020 International Conference on Cyberworlds (CW).

[15] Szu H., Hsu C., Szu C., Wang S. Live Biometric Authenticity Check. Proceedings of the Independent Component Analyses, Wavelets, and Neural Networks.

[16] He Z., Wang W., Dong J., Tan T. (2021). Temporal Sparse Adversarial Attack on Sequence-Based Gait Recognition. arXiv.

[17] Stallings W., Brown L. (2018). Computer Security: Principles and Practice.

[18] Wang M., Abbass H.A., Hu J. Continuous authentication using EEG and face images for trusted autonomous systems. Proceedings of the 2016 14th Annual Conference on Privacy, Security and Trust (PST).

[19] Pham T., Ma W., Tran D., Nguyen P., Phung D. Multi-Factor EEG-Based User Authentication. Proceedings of the 2014 International Joint Conference on Neural Networks (IJCNN).

[20] Poulos M., Rangoussi M., Alexandris N. Neural Network Based Person Identification Using EEG Features. Proceedings of the 1999 IEEE International Conference on Acoustics, Speech, and Signal Processing.

[21] Paranjape R.B., Mahovsky J., Benedicenti L., Koles’ Z. The Electroencephalogram as a Biometric. Proceedings of the Canadian Conference on Electrical and Computer Engineering 2001.

[22] Thomas K.P., Vinod A.P. (2018). EEG-Based Biometric Authentication Using Gamma Band Power During Rest State. Circuits Syst. Signal. Process..

[23] Poulos M., Rangoussi M., Chrissikopoulos V., Evangelou A. Person Identification Based on Parametric Processing of the EEG. Proceedings of the 6th IEEE International Conference on Electronics, Circuits and Systems (Cat. No.99EX357).

[24] Poulos M., Rangoussi M., Alexandris N., Evangelou A. (2002). Person Identification from the EEG Using Nonlinear Signal Classification. Methods Inf. Med..

[25] Hema C.R., Osman A.A. Single Trial Analysis on EEG Signatures to Identify Individuals. Proceedings of the 2010 6th International Colloquium on Signal Processing & its Applications.

[26] Mu Z., Hu J. Research of EEG Identification Computing Based on AR Model. Proceedings of the 2009 International Conference on Future BioMedical Information Engineering (FBIE).

[27] Damaševičius R., Maskeliūnas R., Kazanavičius E., Woźniak M. (2018). Combining Cryptography with EEG Biometrics. Comput. Intell. Neurosci..

[28] Ozdenizci O., Wang Y., Koike-Akino T., Erdogmus D. (2019). Adversarial Deep Learning in EEG Biometrics. IEEE Signal Process. Lett..

[29] Alyasseri Z.A.A., Alomari O.A., Makhadmeh S.N., Mirjalili S., Al-Betar M.A., Abdullah S., Abasi A.K. (2022). EEG Channel Selection for Person Identification Using Binary Grey Wolf Optimizer. IEEE Access.

[30] Thornton C., Hutter F., Hoos H.H., Leyton-Brown K. (2013). Auto-WEKA: Combined Selection and Hyperparameter Optimization of Classification Algorithms. Proceedings of the 19th ACM SIGKDD International Conference on Knowledge Discovery and Data Mining.

[31] Klados M.A., Frantzidis C., Vivas A.B., Papadelis C., Lithari C., Pappas C., Bamidis P.D. (2009). A Framework Combining Delta Event-Related Oscillations (EROs) and Synchronisation Effects (ERD/ERS) to Study Emotional Processing. Comput. Intell. Neurosci..

[32] Palmer J.A., Makeig S., Kreutz-Delgado K., Rao B.D. Newton Method for the ICA Mixture Model. Proceedings of the 2008 IEEE International Conference on Acoustics, Speech and Signal Processing.

[33] Klados M.A., Papadelis C.L., Bamidis P.D. REG-ICA: A New Hybrid Method for EOG Artifact Rejection. Proceedings of the 2009 9th International Conference on Information Technology and Applications in Biomedicine.

[34] Klados M.A., Papadelis C., Braun C., Bamidis P.D. (2011). REG-ICA: A Hybrid Methodology Combining Blind Source Separation and Regression Techniques for the Rejection of Ocular Artifacts. Biomed. Signal. Process. Control.

[35] Delorme A., Makeig S. (2004). EEGLAB: An Open Source Toolbox for Analysis of Single-Trial EEG Dynamics Including Independent Component Analysis. J. Neurosci. Methods.

[36] Pion-Tonachini L., Kreutz-Delgado K., Makeig S. (2019). ICLabel: An Automated Electroencephalographic Independent Component Classifier, Dataset, and Website. NeuroImage.

[37] Hall M., Frank E., Holmes G., Pfahringer B., Reutemann P., Witten I.H. (2009). The WEKA data mining software: An update. ACM SIGKDD Explor. Newsl..

[38] Mu Z., Hu J., Min J. (2016). EEG-Based Person Authentication Using a Fuzzy Entropy-Related Approach with Two Electrodes. Entropy.

[39] Liew S.-H., Choo Y.-H., Low Y.F., Yusoh Z.I.M., Yap T.-B., Muda A.K. (2015). Comparing Features Extraction Methods for Person Authentication Using EEG Signals. Pattern Analysis, Intelligent Security and the Internet of Things.

[40] Zhang S., Sun L., Mao X., Hu C., Liu P. (2021). Review on EEG-Based Authentication Technology. Comput. Intell. Neurosci..

[41] Mao Z., Yao W.X., Huang Y. EEG-based biometric identification with deep learning. Proceedings of the 2017 8th International IEEE/EMBS Conference on Neural Engineering (NER).

[42] Puengdang S., Tuarob S., Sattabongkot T., Sakboonyarat B. EEG-Based Person Authentication Method Using Deep Learning with Visual Stimulation. Proceedings of the 2019 11th International Conference on Knowledge and Smart Technology (KST).

